# Diagnostic ability for common skin diseases among general practitioners working in community health service centers in Shanghai, China: a cross-sectional study

**DOI:** 10.1080/07853890.2024.2442066

**Published:** 2024-12-17

**Authors:** Lei Zhang, Guanghui Wang, Haiying Chen, Xiaoqing Gu, Minjie Jia, Ying Yu, Xiaoxiao Cao, Ruiping Wang

**Affiliations:** a Department of Family Medicine, Xidu Community Health Service Center of Fengxian District, Shanghai, China; b Preventive Health Section, Xidu Community Health Service Center of Fengxian District, Shanghai, China; c Clinical Research & Innovation Transformation Center, Shanghai Skin Diseases Hospital, Medical School, Tongji University, Shanghai, China

**Keywords:** General practitioners, community health service center, skin diseases, diagnostic ability

## Abstract

**Objective:**

Primary care general practitioners (GPs) play a crucial role in common skin diseases (CSDs) diagnosis and treatment for community residents. This study investigates their clinical diagnostic ability for CSDs and influencing factors among GPs in Shanghai.

**Methods:**

In 2023, we recruited 5745 GPs in Shanghai, and online survey was conducted among 5745 GPs with written informed consents. Images of ten CSDs was used to evaluate the diagnostic ability among GPs, logistic regression (LR) analysis was applied to explore influencing factors in GPs with a good skin diseases diagnostic ability.

**Results:**

The 5745 GPs included 1740 male (30.3%), the age ranged from 25 to 60 years with an average of age of 40.8 years. The total score for GSDs diagnostic ability ranged from 2 to 10, with a median of 7.5. In this study, GPs who have achieved ≥ 9 scores were identified as GPs with good diagnostic ability, with a prevalence of 26.7%. LR analysis indicated that GPs with 11 to 15 and over 15 years of work experience had a higher prevalence of good diagnostic ability compared with those with <5 years, the OR was 1.23 (95% CI: 1.02-1.48) and 1.25 (95% CI: 1.05-1.49) respectively. GPs with advanced studies [OR = 1.61, 95% CI:1.33-1.95] and work experience [OR = 1.39, 95% CI:1.20-1.61] in dermatology had a higher prevalence of good diagnostic ability.

**Conclusion:**

The diagnostic ability for CSDs was good among GPs in Shanghai. GPs with more years of work, advanced studies and work experience in dermatology have better diagnostic abilities CSDs.

## Introduction

Skin diseases is a common reason for primary care visits among community residents [1–3]. Chronic skin problems place heavy burden on patients, including the impaired health-related quality of life, decreased occupational productivity, and severe psychological effects [4,5]. Moreover, the adverse health effects of skin disorders not only include the loss of body function, but also impose heavy economic burden on their family and the society, or even death [6]. So, skin condition is an important public health issue and the fourth leading cause of non-fatal disease burden worldwide [7].

Currently, the development of competence in skin diseases diagnosis and treatment among general practitioners has gained global attention, and it is recommended to appropriately increase the content of dermatology-related education and training among general practitioners at the medical student stage [1,8]. Skin diseases are common health problems among community residents, and the need for skin diseases diagnosis and treatment is growing rapidly in primary healthcare service among community residents. However, general practitioners working in community health service centers are not well skilled in the diagnosis and treatment of skin diseases due to many conditions which involving limited expertise and the lack of professional and specialized training [9–12]. So general practitioners need to be trained to improve and strengthen their ability to diagnose and treat skin diseases. In recent years, the Expert Group of Guidelines for Primary Care of Skin and Venereal Diseases of the Chinese Medical Association (CMA) has issued several guidelines for skin diseases primary care improving and standardizing the diagnosis and treatment of common skin diseases among general practitioners [13–18].

In China, with the deepening reform of healthcare systems and the establishment of a perfect system of hierarchical diagnosis and treatment, governments at all levels have continued to enhance the capacity of community health services and give full play to the fundamental role of community health services in the healthcare service system. Shanghai government announced the ‘List of Basic Diseases in Shanghai’s Community Healthcare Services’ in 2023, which requires the primary healthcare institutions should constantly incorporate a variety of specialized services at the grass-roots level in the community. So the general practitioners should provide medical services such as initial diagnosis, routine treatment, and serious diseases referral service for patients with skin diseases. However, evidence regarding the diagnosis and treatment of common skin diseases among the general practitioners working in community health service centers in Shanghai remains limited. We conducted this cross-sectional survey to understand the current status of the clinical diagnosis of common skin diseases and potential influencing factors among general practitioners working in community health service centers in Shanghai, China, and to provide guidance for future intervention and specialized training.

## Methods

### Study population

This study was conducted from January to June 2023 in Shanghai, China, and all of the two hundred and forty community healthcare service centers in Shanghai were invited to participate in the study. Quota sampling combined with random sampling were used to select the general practitioners (GPs) from the 240 community health service centers. Firstly, a list of all general practitioners in each community health service centers was pre-arranged and set as the sample frame. Secondly, 25 GPs were randomly selected from each of community health service centers based on the sample frame, and 6000 GPs were finally selected for this survey. An online survey was implemented and 5745 out of the 6000 selected GPs completed the questionnaire with a response rate of 95.75%. The written informed consents were obtained from all participants prior to their online questionnaire survey. This study was reviewed and approved by the Institutional Review Board of Shanghai Skin Diseases Hospital, Shanghai, China (2022-31).

### Data collection

**Figure 1.**
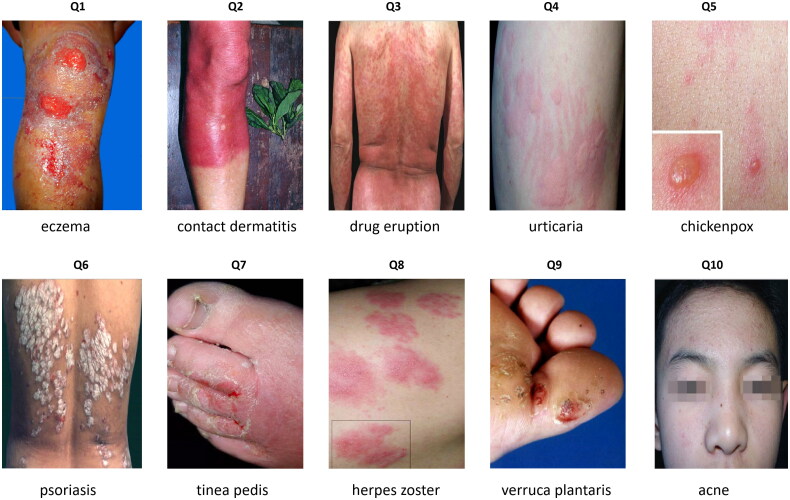
Images of 10 common skin diseases used to assess the diagnostic ability of general practitioners in community health service centers in Shanghai, China.

In this study, the online survey with structured questionnaire was used for data collection. The questionnaire included three parts: (1) demographic information (age, gender, education level, marital status, etc.); (2) work experience (years of work, workplace (urban/suburban), the standardized and specialty training experience, dermatology refresher experience, and dermatology work experience, etc.); and (3) 10 images of common skin diseases used to evaluate the diagnostic ability among GPs, involving eczema, contact dermatitis, drug eruption, urticaria, chicken pox, tinea pedis, herpes zoster, verruca plantaris, and acne (Figure 1). For each image of the 10 common skin diseases, four different answer options were provided with only one option being correct. Moreover, We confirm that the written consent to publish these 10 images have been obtained from the 10 images related patients in this study.

### Definition and index calculation

In this study, we scored 1 for the correct diagnosis and 0 for the incorrect diagnosis to each of the 10 images of common skin diseases, and then accumulated them together as the total score to display the diagnostic ability for common skin diseases. So the total score for common skin diseases diagnosis ranged from 0 to 10, and we defined a GP with a good diagnostic ability for common skin diseases as those who scored at least 9 of the 10 total scores. In this study, we categorized the age of the participants as <30 years, 30-40 years, 41-50 years, >50 years, and years of work into <5 years, 5-10 years, 10-15 years, >15 years. Education was recorded as the number of years of schooling completed and was categorized as 0-9 years (junior high school and lower), 10-12 years (senior high school), and >12 years (college and above).

### Data analysis

Data were analyzed using SPSS (version 26; SPSS Inc., Chicago, Illinois, United States). Quantitative variables were expressed as mean and standard deviation (SD) with normal distribution, and t-tests were used for the comparisons between groups. Quantitative variables were expressed as median and interquartile range (IQR) with skewed distribution, and rank sum tests were used for the comparisons between groups. Qualitative variables were expressed as frequency counts (n) and percentage (%), and the chi-squared test was used for comparison between groups. Logistic regression model was used to calculate the odds ratios (OR) and 95% confidence intervals (CI) to explore the influencing factors among GPs with good skin diseases diagnostic ability. In this study, a p-value of <0.05 (two-tailed) was considered statistically significant.

## Results

In this study, 5745 GPs included 1740 male (30.3%), with ages ranging from 25 to 60 years, with an average age of 40.8 years (SD: 8.2 years). In this study, over 68% of the 5745 GPs worked in the suburban districts, and 80% and 14% of them had an education of undergraduate or master and above education, respectively. The median number of years worked was 10 (IQR: 6-16 years), and nearly 60% of GPs had experience in standardized training for general practice, but only 9% and 18% of them had advanced studies and work experience in dermatology, respectively. Moreover, nearly half of the GPs had work experience in general hospitals. In this study, female GPs were younger than male GPs, and female GPs also had a higher education level, higher proportion of standardized training for general practice, but fewer year of work experience, and a lower proportion of the advanced studies in dermatology or work experience in dermatology than males, the differences were all statistically significant (*p* < 0.05), Table 1.

**Table 1.** Demographic feature of general practitioners in community health service center in Shanghai, China.

Variables	Total GP (*n* = 5745)	Male GP (*n* = 1740)	Female GP (*n* = 4005)	t/χ^2^	P
Age (years), mean (SD)	40.8 (8.2)	41.9 (8.6)	40.3 (8.1)	6.68	<0.01
Age (years), n (%)				45.81	<0.01
<30	562 (9.8)	144 (8.3)	418 (10.4)		
30-40	1940 (33.8)	500 (28.7)	1440 (35.9)		
41-50	2341 (40.8)	773 (44.4)	1568 (39.2)		
>50	902 (15.7)	323 (18.6)	579 (14.5)		
Work district, n (%)				3.08	0.08
Downtown district	1824 (31.8)	524 (30.1)	1300 (32.5)		
Suburb district	3921 (68.3)	1216 (69.9)	2705 (67.5)		
Education, n (%)				81.03	<0.01
Junior college	348 (6.1)	156 (9.0)	192 (4.8)		
Undergraduate	4610 (80.2)	1432 (82.3)	3178 (79.4)		
Master and above	787 (13.7)	152 (8.7)	635 (15.9)		
Years of work, median (IQR)	10.0 (6.0-16.0)	12.0 (7.0-18.0)	10.0 (5.0-16.0)	37.65	<0.01
Year of work (years), n (%)				58.36	<0.01
<5	11009 (17.6)	255 (14.7)	754 (18.8)		
5-10	1266 (22.0)	338 (19.4)	928 (23.2)		
11-15	1400 (24.4)	395 (22.7)	1005 (25.1)		
>15	2070 (36.0)	752 (43.2)	1318 (32.9)		
Standardized training for GP, n (%)				13.75	<0.01
Yes	2406 (58.1)	665 (38.2)	1741 (43.5)		
No	3339 (41.9)	1075 (61.8)	2264 (56.5)		
Advanced study in dermatology, n (%)				14.11	<0.01
Yes	508 (8.8)	191 (11.0)	317 (7.9)		
No	5237 (91.2)	1549 (89.0)	3688 (92.1)		
Work experience in dermatology, n (%)				26.18	<0.01
Yes	1026 (17.9)	379 (21.8)	647 (16.2)		
No	4719 (82.1)	1361 (78.2)	3358 (83.8)		
Work experience in hospitals, n (%)				0.53	0.47
Yes	2736 (47.6)	816 (46.9)	1920 (47.9)		
No	3009 (52.4)	924 (53.1)	2085 (52.1)		

SD: standard deviation.

IQR: inter-quartile range.

CHSC: community health service center.

GP: general practitioner.

### Diagnostic ability for common skin diseases in GPs

**Figure 2.**
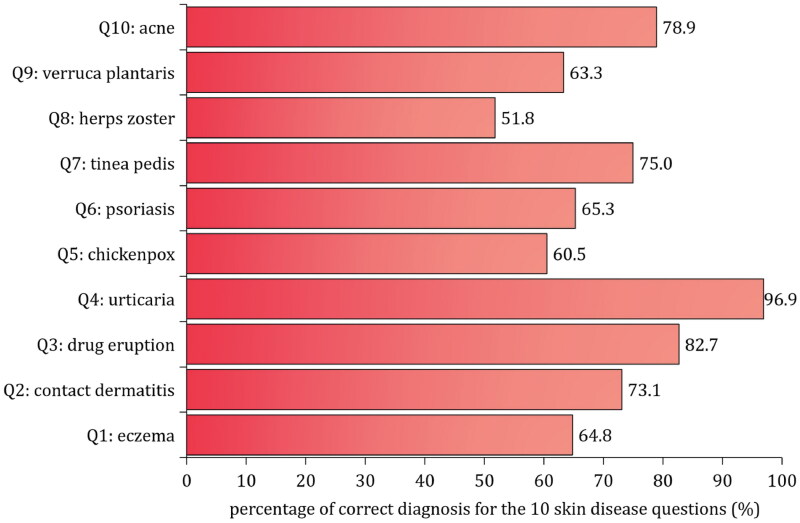
Percentage of correct diagnoses for the 10 common skin diseases among 5745 general practitioners in community health service centers in Shanghai, China.

**Figure 3.**
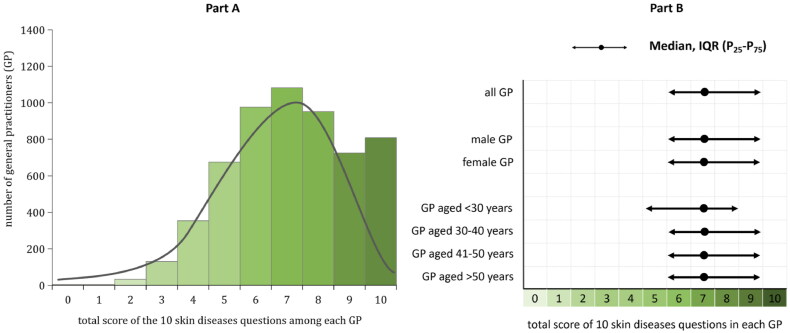
The bar chart of the total score for the 10 skin diseases questions among the 5745 general practitioners (GP) and the median (IQR) value of the total score among all GP and GP by different gender and age as well in Shanghai, China.

Figure 2 shows the percentage of correct diagnosis for each of the 10 common skin diseases among GPs. The percentage of correct diagnosis was all over 50% for each of the 10 common skin diseases, with the lowest correct diagnosis percentage of 51.8% for herpes zoster, and the highest correct diagnosis percentage of 96.9% for urticaria. The total score for skin diseases diagnosis evaluation among the 5745 GPs ranged from 2 to 10, with a median total score of 7.5 (IQR: 6-9). Data in Figure 3 part B indicated that the median value of the total score for skin diseases diagnosis was 7.5 (IQR: 6-9) in male GPs which was the same in female GPs (median value = 7.5, IQR: 6-9). Moreover, the median value of the total score for skin diseases diagnosis was 7.5 (IQR: 6-9) for GPs in each of the four different age groups (Figures 2 and 3).

### Factors associated with the good diagnostic ability of skin diseases in GPs

In this study, 1534 out of the 5742 GPs who achieved at least 9 scores were identified as GPs with good diagnostic ability for common skin diseases, the prevalence was 26.7%. Chi-square tests indicated that GPs aged over 50 years had a higher prevalence of good diagnostic ability than those aged 41-50, 30-40 and <30 years, and logistic regression indicated that GPs aged over 50 years had higher prevalence of good diagnostic ability [OR = 1.25, 95%CI: 1.00-1.59] than those aged <30 years. The prevalence of good diagnostic ability among GPs with over 10 years of work experience was higher, and logistic regression analysis indicated that GPs with 11-15 and over 15 years of work experience had higher prevalence of good diagnostic ability of skin diseases than those with less than 5 years, the OR was 1.24 (95%CI: 1.03-1.49) and 1.26 (95%CI: 1.06-1.50) respectively. GPs with advanced study in dermatology [OR = 1.73, 95% CI:1.43-2.09], and with work experience in dermatology [OR = 1.47, 95% CI:1.27-1.70] also had higher prevalence of good diagnostic ability of skin diseases (Table 2).

**Table 2.** The proportion of general practitioners (GP) who have achieved at least 9 scores out of the 10 skin diseases questions and its associated influencing factors among the 5745 GP in Shanghai, China.

Variables	Proportion of PG achieved ≥90% scores, n(%)	Model A		Model B		Model C	
		OR	95% CI	OR	95% CI	OR	95% CI
Age ǂ							
<30	136 (24.2)	1.00	–	1.00	–		
30-40	494 (25.5)	1.07	0.86-1.33	0.97	0.76-1.24		
41-50	647 (27.6)	1.20	0.97-1.48	1.06	0.82-1.39		
>50	257 (28.5)	***1.25***	***1.00-1.59***	1.07	0.79-1.44		
Year of work (years) ǂ							
<5	242 (23.9)	1.00	–	1.00	–	1.00	–
5-10	311 (24.6)	1.03	0.85-1.25	1.01	0.83-1.25	1.01	0.84-1.23
11-15	393 (28.1)	***1.24***	***1.03-1.49***	1.20	0.97-1.49	***1.23***	***1.02-1.48***
>15	588 (28.4)	***1.26***	***1.06-1.50***	1.18	0.94-1.48	***1.25***	***1.05-1.49***
Advanced study in dermatology ǂ							
Yes	190 (37.4)	***1.73***	***1.43-2.09***	***1.62***	***1.33-1.96***	***1.61***	***1.33-1.95***
No	1344 (25.7)	1.00	–	1.00	–	1.00	–
Work experience in dermatology ǂ							
Yes	341 (33.2)	***1.47***	***1.27-1.70***	***1.39***	***1.20-1.62***	***1.39***	***1.20-1.61***
No	1193 (25.3)	1.00	–	1.00		1.00	
Gender							
Male	449 (25.8)	1.00	–				
Female	1085 (27.1)	1.07	0.94-1.21				
Work district							
Downtown district	497 (27.3)	1.00	–				
Suburb district	1037 (26.5)	0.96	0.85-1.09				
Education							
Junior college	102 (29.3)	1.00	–				
Undergraduate	1227 (26.6)	0.88	0.69-1.12				
Master and above	205 (26.1)	0.85	0.64-1.12				
Standardized training for GP							
Yes	647 (26.9)	1.01	0.90-1.15				
No	887 (26.6)	1.00	–				
Work experience in hospitals						1	
Yes	718 (26.2)	0.96	0.85-1.08				
No	816 (27.1)	1.00	–				

ǂ: the differences between groups was statistically significant (*p* < 0.05).

Model A: Uni-variate logistic regression to explore factors associated with GP who achieved at least 9 out of 10 total scores in Shanghai.

Model B: Multi-variate logistic regression to explore factors associated with GP who achieved at least 9 out of 10 total scores with the mutual adjustment of age, year of work, experience of advanced study in dermatology, and work experience in dermatology in Shanghai.

Model C: Multi-variate logistic regression to explore factors associated with GP who achieved at least 9 out of 10 total scores with the mutual adjustment of year of work, experience of advanced study in dermatology, and work experience in dermatology in Shanghai.

OR: odds ratio.

CI: confidence interval.

GP: general practitioner.

The Multivariate logistic regression analysis with the adjustment of potential influencing factors in Model B and Model C indicated that GPs with 11-15 and over 15 years of work experience had a higher prevalence of good skin diseases diagnostic ability than those with less than 5 years, the OR was 1.23 (95% CI: 1.02-1.48) and 1.25 (95% CI: 1.05-1.49) respectively. GPs with advanced studies in dermatology [OR = 1.61, 95% CI:1.33-1.95], and work experience in dermatology [OR = 1.39, 95% CI:1.20-1.61] had a higher prevalence of good diagnostic ability for skin diseases (Table 2).

### Association between good diagnostic ability of skin diseases and work years in GPs

**Figure 4.**
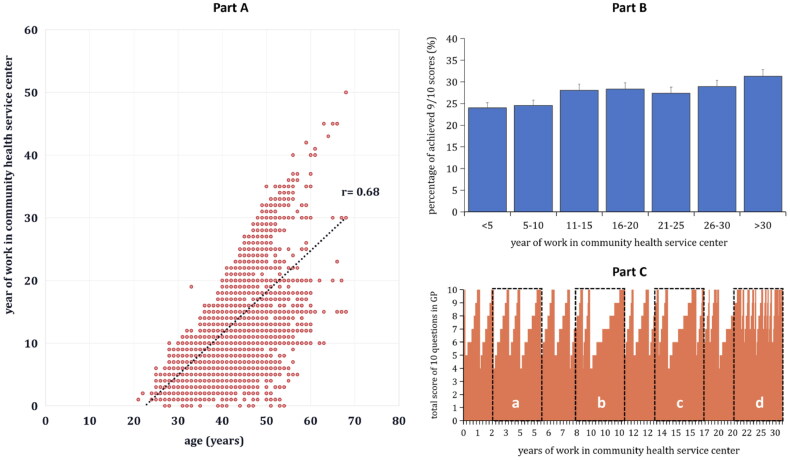
Scatter plot exploring the correlation between age and years of work in community health service centers (CHSC) among 5745 general practitioners (GPs) (Part A), percentage of GPs achieving at least 9 out of 10 correct diagnoses for the 10 skin diseases by years of work (Part B), and the total score distribution for each GP (Part C) in Shanghai, China.

Part A in Figure 4 indicates that years of work experience was positively associated with age among general practitioners (rs = 0.68, *p* < 0.01). To explore the association between the prevalence of good diagnostic ability for skin diseases and year of work in depth, we categorized the years of work into seven groups, including <5 years, 5-10 years, 11-15 years, 16-20 years, 21-25 years, 26-30 years, and >30 years. Part B in Figure 4 shows that the prevalence of good diagnostic ability for common skin diseases increased gradually with an increase in work experience (χ^2^ for trend = 6.32, *p* < 0.05). Moreover, the individual level of good diagnostic ability (achieved at least 9 out of 10 scores for the 10 common skin diseases images) also demonstrated that for the same number of GPs (such as GP in frames a, b, c and d), the number of GPs with good skin diseases diagnostic ability increased with an increase in years of work experience (Figure 4).

## Discussion

In this study, we assessed the common skin diseases diagnostic ability among the general practitioners in Shanghai and explored factors influencing their correct diagnostic ability for skin diseases. Findings indicated that the GPs working in community health service centers in Shanghai had good diagnostic ability for common skin diseases and over a quarter of GPs have achieved a score of at least 9 out of 10 score regarding the 10 common skin diseases. Moreover, GPs with more work years, with advanced study and work experience in dermatology had better skin diseases diagnostic ability.

In this study, GPs in community health service centers generally had good skin diseases diagnostic ability, with a median value of 7.5 for the total score of correct diagnosis. These findings are in line with a study conducted by Hatem M Alotaibi in Saudi region [19]. In Shanghai, the GPs are required to undergo a 3-year of standardized training program as resident before they can work in primary care communities. The standardized training program was usually implemented by the tertiary hospitals affiliated the medical school of universities, and the contents of training program involving of dermatology, ophthalmology, stomatology, etc. Therefore, the solid, standardized and long-term training program could ensures GPs in Shanghai to achieve a good ability in the diagnosis of common skin diseases, even among GPs working in the community health service centers.

GPs in the community health service centers usually confront with a wide range of skin diseases consultation. Due to common skin diseases are generally had similar clinical features, it is crucial for GPs to correctly identify and diagnose these skin diseases before providing treatment [1,19–21]. In this study, 10 common skin diseases were selected to assess the correctly diagnostic ability among GPs. The findings indicated that the percentage of correct diagnosis for acne, tinea pedis and drug eruption was all over 75%, and the percentage of correct diagnosis was 96.9% for urticaria. However, the percentage of correct diagnosis for herpes zoster was only 51.8%. Previous studies reported that the incidence of herpes zoster was (2.9-5.8) per1000 person-years among people aged ≥50 years in China [22]. Herpes zoster is usually accompanied by neuropathic pain except for skin lesions, which seriously affects the quality of life of patients. So the timely targeted antiviral therapy could help skin lesions heal quicker and may shorten the diseases duration of herpes zoster pain [23]. However, herpes zoster is more difficult to diagnose when there are no lesions but only pain during the prodromal phase. In some cases, patients present with a rash-less form of herpes zoster, known as ‘zoster sine herpete’, which is more painful and lasts longer than typical herpes zoster [24]. Also, in the elderly, immunocompromised patients, and pregnant women, the lesions are often atypical, further complicating the diagnosis. In this study, the lower percentage of correct diagnosis for herpes zoster may also due to the fact that most of the GPs in community health service centers in Shanghai were not underwent the speciality training for dermatology, so they may encounter challenges in diagnosis of the herpes zoster, particularly in those atypical patients and immunocompromised patients as well as pregnant women. Therefore, In our subsequent efforts, we will target GPs to improve their competence in the management of skin diseases, and focusing on the training of differential diagnosis and treatment of atypical herpes zoster in future training and clinical practice among GPs in Shanghai, China.

In this study, we noticed that GPs with advanced dermatology training experience and dermatology work experience had a significantly higher positive impact on their performance in common skin diseases recognition and diagnosis. A study conducted by TP Lam in Hong Kong demonstrated that a short dermatology training course for primary care general practitioners could significantly improve their confidence, attitudes, and skills in the diagnosis and management of common skin diseases. The findings of the Hong Kong study also indicated that GPs with short dermatology training course could promote more patients with skin diseases being managed in practice, resulting in fewer patients being referred to higher-level hospitals [25]. So we recommend that on the-job training specialized in common skin diseases education should be strengthened and implemented among GPs to improve their ability of skin diseases identification and diagnosis, especially among those working in community health server centers.

This study also demonstrated that work year affected the performance of general practitioners in the identification and diagnosis of common skin diseases. In this study, the total score for skin diseases correct diagnosis increased with the increase in year of working experience, which was in line with previous studies [19]. Medicine is an empirical discipline, with the accumulation of work years, GPs could achieve deeper knowledge and understanding for skin diseases, and was disciplined to gain more and comprehensive diagnostic skills through clinical practice. Therefore, GPs with more years of working experience tended to perform well in skin diseases diagnosis and verification.

In China, GPs working in the community health service centers are responsible for common skin diseases diagnosis and treatment. Meanwhile, the GPs in China also play the role of a bridge to connect community health service centers with tertiary hospitals, and provide bidirectional referrals for patients with medical needs, which is similar to Europe and the United States [26]. In spite the broad availability of resources such as dermatology books, online images, and on-duty training opportunities, GPs working in community health service centers still have lower diagnostic ability for skin diseases than specialized dermatologists. This might be due to the limited clinical practice and lack of specialized dermatology training opportunities [11,27]. Previous studies indicated that short-term training can improve the ability to recognize and diagnose common skin diseases and could lead to a greater improvement in career competence among GPs [28–31]. So we should provide more training focusing on skin diseases diagnosis and treatment among GPs in the future.

In this study, despite of the high total score for skin diseases diagnosis achieved among GPs working in community health service centers, we should note that there was still a quarter of the GPs achieved less than 5 out of the total 10 scores, especially for herpes zoster, chickenpox and verruca plantaris. Therefore, training programs should be implemented to improve diagnosis and treatment ability. Firstly, more attention should be paid to the standardized training program focusing on skin diseases and training programs should be implemented by efficient organization [32]. Secondly, GPs should make full use of expert consensus and guidelines which provide concise diagnostic tools and evidence-based referral recommendations to facilitate the use of primary care in clinical practice. Thirdly, it is recommended that the Medical Association conduct regular research to understand the capacity and level of service for common skin diseases in primary care organizations and provide online precision training to address these weaknesses. Finally, it is recommended to establish and improve the remote dermatology diagnosis and treatment platforms which will facilitate the in time learning from the approaches implemented in other regions [33].

This study has some limitations. Firstly, the GPs in study enrolled in the study were from the 240 community health service centers in Shanghai, which ensures the high internal authenticity, however, the generalization of the findings to represent the whole story in China is limited. Secondly, the skin diseases diagnostic ability was assessed by referring 10 images of common skin diseases without providing additional clinical information, which might have lead to information bias. Thirdly, we only included years of working experience, advanced training experience in dermatology and work experience in dermatology to assess their influence on skin disease diagnosis among GPs, but more factors need to be considered in the future, such as speciality in college discipline, frequency of training received, and so on. Fourthly, only 10 images of common skin diseases were included in this study, which was not comprehensive to represent the overall diagnosis ability of common skin diseases in GPs, the incorporation of more skin diseases including skin cancer should be considered. Finally, the main conclusion of this study only reflect the visual diagnostic competence of general practitioners for common skin disorders and do not involve an assessment of therapeutic competence. Therefore, incorporating improvements should be considered in future studies.

## Conclusions

The overall diagnostic ability for common skin diseases was good among GPs working in the community health service centers in Shanghai. GPs with more years of work experience, advanced studies and work experience in dermatology had better diagnostic abilities. So we recommend to implement the specialized training programs focusing on common skin diseases diagnosis and treatment among GPs, especially among those with few years of work experience and those lacking advanced training.

## Data Availability

The data for this study are available upon request from the corresponding author. The request should state the title and aim of the research for which the data are requested.
